# The Influence of Shyness on the Scanning of Own- and Other-Race Faces in Adults

**DOI:** 10.1371/journal.pone.0052203

**Published:** 2012-12-19

**Authors:** Qiandong Wang, Chao Hu, Lindsey A. Short, Genyue Fu

**Affiliations:** 1 Department of Psychology, Zhejiang Normal University, Jinhua, Zhejiang, China; 2 Department of Human Development and Applied Psychology, University of Toronto, Toronto, Ontario, Canada; 3 Department of Psychology, Brock University, St. Catharines, Ontario, Canada; 4 Hangzhou College for Kindergarten Teachers, Zhejiang Normal University, Hangzhou, Zhejiang, China; University of Leicester, United Kingdom

## Abstract

The current study explored the relationship between shyness and face scanning patterns for own- and other-race faces in adults. Participants completed a shyness inventory and a face recognition task in which their eye movements were recorded by a Tobii 1750 eye tracker. We found that: (1) Participants’ shyness scores were negatively correlated with the fixation proportion on the eyes, regardless of the race of face they viewed. The shyer the participants were, the less time they spent fixating on the eye region; (2) High shyness participants tended to fixate significantly more than low shyness participants on the regions just below the eyes as if to avoid direct eye contact; (3) When participants were recognizing own-race faces, their shyness scores were positively correlated with the normalized criterion. The shyer they were, the more apt they were to judge the faces as novel, regardless of whether they were target or foil faces. The present results support an avoidance hypothesis of shyness, suggesting that shy individuals tend to avoid directly fixating on others’ eyes, regardless of face race.

## Introduction

It is possible to infer an individual’s emotional state, age, sex, race, and other identity-relevant information simply by looking at his or her face [1]. Hence, scanning others’ faces is extremely important for our understanding of others and plays a vital role in our everyday social interactions. It is therefore not surprising that a network of areas in the human lateral middle fusiform gyrus [2] and inferior occipital cortex [3] respond preferentially to faces. In recent years, advances in eye-tracking technology have allowed for increasingly precise measurements of adults’ gaze patterns while viewing such socially relevant stimuli as human faces and have provided insight into the attentional mechanisms that underlie complex face scanning.

Considerable research has examined the influence of culture on face scanning [4], [5] and on facial expression scanning [6]. However, relatively few studies have examined individual differences in face processing [7] and the way in which individuals’ personality characteristics influence identity recognition and facial scanning patterns. Personality traits may particularly affect facial scanning patterns because such characteristics shape aspects of social cognition as basic as eye contact [8]. For example, both shyness and social anxiety, terms that have been used interchangeably in previous literature despite having slightly different meanings [9], [10], are characterized by a preoccupation of the self when engaging in or anticipating a real or imagined social situation [11]. Accordingly, one highly salient feature of shyness and social anxiety is gaze aversion and the avoidance of face-to-face interaction [12].

In social interactions, non-clinically socially anxious individuals make less eye contact with an interviewer than do their non-socially anxious counterparts [13]. Furthermore, increased social anxiety has been related to decreased eye contact [14]; however, this relationship has not been observed in all studies [15].

Recent research has used eye-tracking technology to explore the relationship between shyness and face scanning patterns; however, results have been inconsistent. Horley, Williams, Gonsalvez, and Gordon [16] found that socially phobic individuals tend to avoid fixating on the eye region when asked to scan emotional faces and that such avoidance is particularly pronounced for sad faces. In contrast, Wieser, Pauli, Alpers, and Mühlberger [17] demonstrated that women with high anxiety look longer than non-socially anxious individuals at the eye region of the face, regardless of whether the direction of gaze is direct or averted. Similarly, Brunet, Heisz, Mondloch, Shore, and Schmidt [18] found that among children, shyness is associated with longer gaze duration at the eye region relative to the mouth region, suggesting that shy children do not avoid looking at the eyes. The discrepancy between past studies may be due to the different subject samples, different experimental paradigms, or different areas of interest (AOIs) defined across studies.

The primary goal of the present study was to examine the relationship between shyness and face scanning among adult participants and to extend on previous research in two ways. First, we used both own- and other-race test faces to examine whether the relationship between shyness and face scanning differs among faces of different races. Although some researchers have found that participants use the same eye movement strategies when scanning own- and other-race faces [4], [5], Fu, Hu, Wang, Quinn, and Lee [19] recently demonstrated that race of face can influence participants’ scanning patterns such that Chinese participants spend increased time looking at the eye region of Caucasian faces relative to Chinese faces and increased time looking at the nose and mouth of Chinese faces relative to Caucasian faces. Second, we used iMap Matlab toolbox to examine the location of significant fixations, a novel method that computes statistical fixation maps of eye movements [20]. Unlike the AOI analyses that amalgamate all fixation points that fall into a particular predetermined area of interest and then perform statistical tests on the total fixations to the area between conditions, iMap allows for statistical testing of condition differences on any part of a stimulus without the restriction of the AOIs and for statistical testing of condition differences on a scale finer than that of the AOI analyses. Such a technique allows for a more fine-grained and detailed analysis of participants’ scanning patterns relative to the methods employed in past studies.

We hypothesized that participants’ shyness levels would affect scanning patterns in face recognition such that increased shyness would be associated with decreased time spent fixating on the eyes. Additionally, we hypothesized that race of face would moderate the magnitude of this effect such that the lack of visual experience with other-race faces would diminish the effect.

## Method

### Ethics Statement

**Figure 1:**
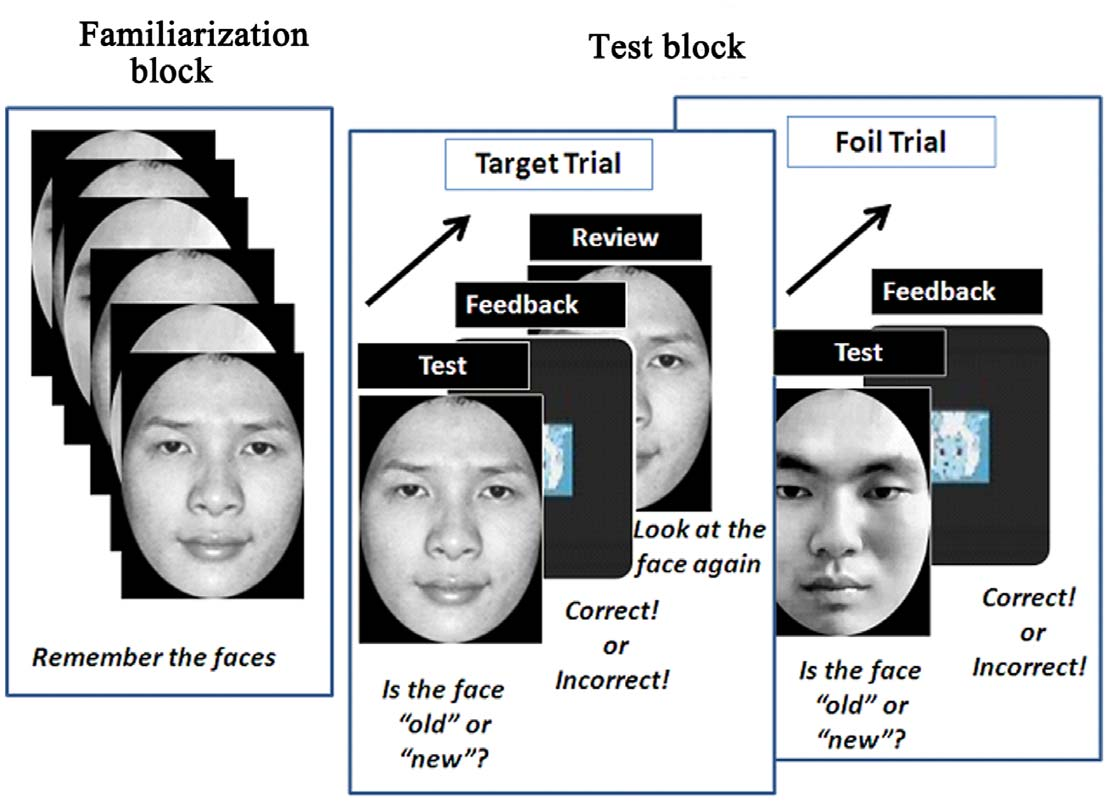
A schematic representation of the experimental design.

**Figure 2:**
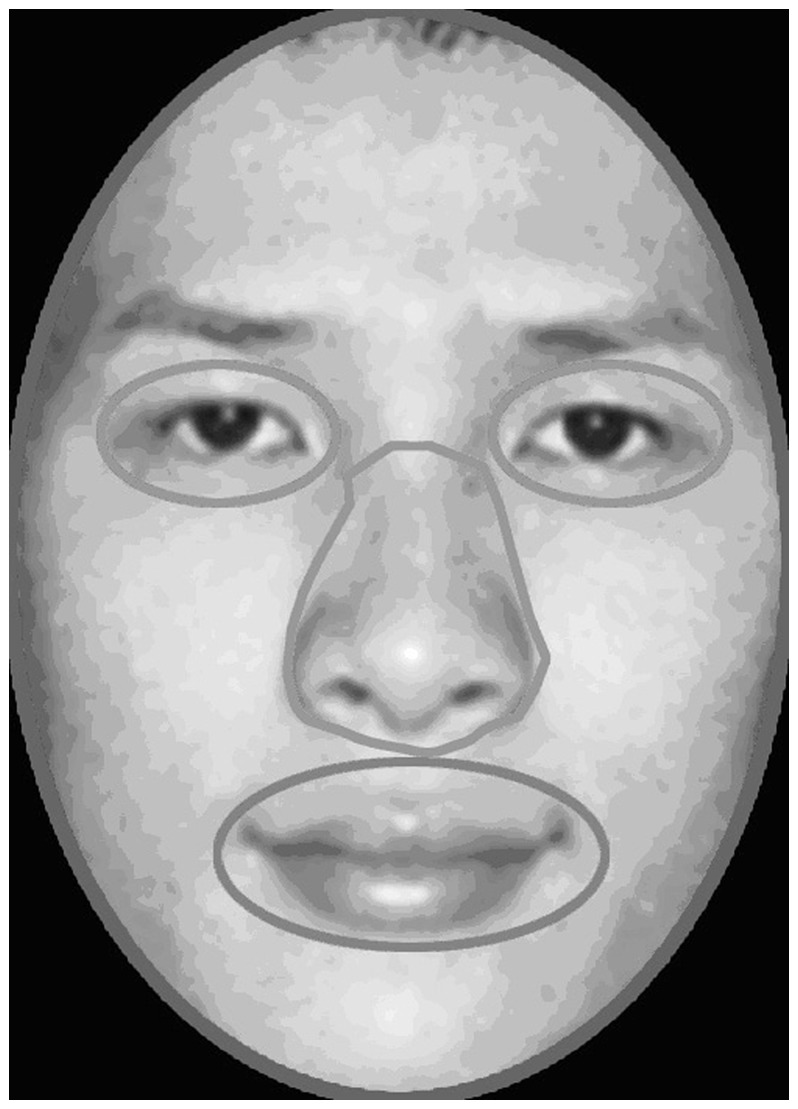
Sample area of interest (AOI) plot.

**Figure 3:**
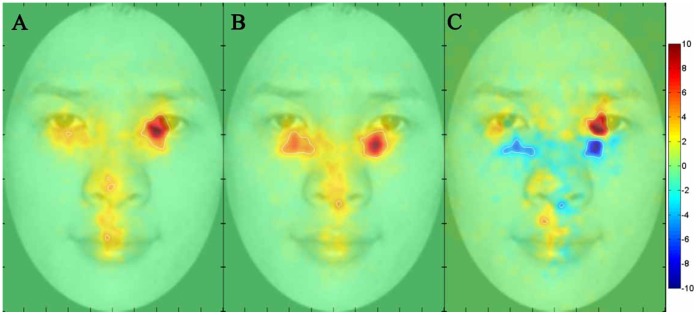
Fixation maps for the low and high shyness participants in the learning and reviewing phase. **A**) Fixation map for the low shyness participants, **B**) Fixation map for the high shyness participants, and **C**) Fixation difference map calculated by subtracting the high shyness group from the low shyness group.

**Figure 4:**
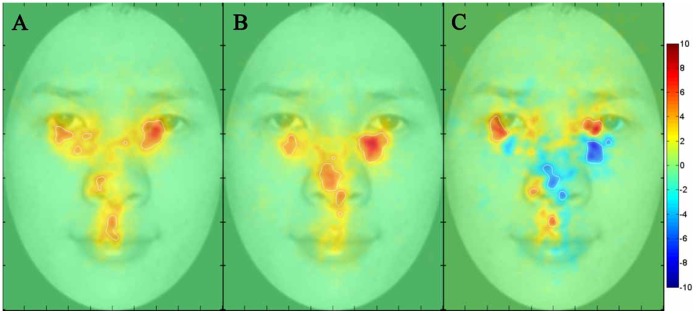
Fixation maps for the low and high shyness participants in the recognition phase. **A**) Fixation map for the low shyness participants, **B**) Fixation map for the high shyness participants, and **C**) Fixation difference map calculated by subtracting the high shyness group from the low shyness group.

This study was conducted in China according to the NIH research ethics guidelines and received approval from the Zhejiang Normal University Research Ethics Review Committee. Participants gave written informed consent prior to their participation and were compensated for their involvement in the study. Participants were ensured that no harm would come to them through their involvement in the study and were told that they had the option to quit at any time during the experiment and still receive monetary payment. The subjects of the images used in Figures 1, 2, 3, and 4 of this manuscript provided written informed consent, as outline in the PLOS consent form, to publication of their photograph.

### Participants

Thirty right-handed Chinese undergraduate students from Zhejiang Normal University (12 male; Mean age = 20.17 years, *SD* = 1.28 years) participated in this experiment. One male participant was not included in the final data analysis due to computer failure.

### Materials

Twenty Chinese face photographs (10 male), twenty African-American face photographs (10 male), and twenty South Asian (e.g., Indian, Pakistani) face photographs (10 male) were used as stimuli. All photographs were standardized at 500 pixels (13.5 cm; 12.7 degrees of visual angle) wide and 700 pixels (18.9 cm; 17.9 degrees of visual angle) high and had a resolution of 72 pixels per inch. All face images were shown in frontal view and rendered in grey scale to prevent differences in skin tone between the different face races from affecting participants’ scanning of the images. To further control for hairstyle differences, all face images were overlaid with the same elliptical shape. Furthermore, to control for the influence of participants’ bottom-up processing related to low-level stimulus attributes such as luminance and contrast [21], the images were matched in overall brightness and luminance using Shine Matlab toolbox.

A Tobii 1750 eye tracker (0.5 degree precision, 17 inch, 50 Hz sample rate, 5 fps per second, 1280×1024 pixels resolution) was used to record participants’ fixations on the face images. The Tobii Studio program was used to control the stimulus presentation.

The 13-item revised Cheek and Buss Shyness Scale [22] was used to measure participants’ level of shyness. This scale has been previously shown to be both highly reliable and valid among Chinese participants, with a Cronbach alpha of.90 and test-retest reliability of 0.88 [23]. A sample item from the shyness inventory includes the following statement: “I feel nervous when I stay with a person with whom I am not familiar.”

### Procedure

Participants completed the study individually. They were positioned 60 cm from the eye tracker screen and used a mouse connected to the computer running the Tobii Studio program to respond. Response time and accuracy rates were recorded by the Tobii Studio program. Fixation data were recorded by the eye tracker automatically. Participants first completed a practice phase. Four images (four Caucasian faces, two per gender) were presented one at a time, and participants were instructed to remember each face. The old faces were then mixed with four novel faces (four Caucasian faces, two per gender), and upon seeing each face, participants judged whether the image was old or new. All participants received a perfect score during the practice phase and thus none were excluded due to a failure to understand the experimental task.

Before the experimental phase, the eye movements of participants were calibrated. The calibration program asked participants to follow a bouncing red dot with their eyes as it moved around the screen. The diameter of the red dot periodically changed from 0 to 1 inch. If a participant’s fixation was more than 1 inch away from the center of the dot, a recalibration was performed. Once the calibration was successful, the familiarization block (Block 0) of the experimental phase began. The results of this calibration were used to calculate the fixation points of the participants in the familiarization block.

The experimental phase consisted of one familiarization block (Block 0) and four test blocks (Blocks 1–4). In the familiarization block, participants were shown 12 target faces (four Chinese, four South Asian, and four African-American; two males per race). The faces were randomly chosen from the set of 60 faces. Each face was shown for 3 seconds followed by a cartoon character used as a mask (2 seconds). The cartoon character also verbally announced, “The next image” over a loudspeaker after the presentation of each face.

After all 12 images were presented, the experimenter initiated the first test block. At the beginning of this test block, the aforementioned calibration procedure was run again and the result of this calibration was used to calculate the fixation points of the participants in the first test block. Following the calibration, the familiarized target face (old face) or a new foil face was shown. Participants were instructed to respond as quickly and as accurately as possible by pressing a key to indicate whether it was an old or new face (Test). As soon as participants responded, the cartoon character appeared for 2 seconds, verbally announcing whether the face was an old or new face to give participants feedback (Feedback). If the preceding face was a familiarized target face, the cartoon face reappeared and announced that the face was an old face and would be shown again, after which the target face just seen would be shown for 3 seconds for participants to review (Review) before the next trial began. We used this test-feedback-review cycle in order to gain additional eye movement data regarding the encoding of familiarized faces. If the preceding face was a new foil face, the cartoon character appeared for 2 seconds, announcing that the face was a new face (Feedback) but the foil face would not be reviewed. Immediately, the cartoon character announced, “The next image,” after which a new trial began. This test-feedback-review (test-feedback) cycle was repeated until all 12 target faces and all 12 foil faces were shown (24 trials in total). The foil faces included four Chinese, four South Asian, and four African-American faces (two males per race) that were never used as familiarization faces. The order in which the target and foil faces were shown was randomized.

Once the first block was completed, participants were given a break for approximately 1 minute to avoid fatigue. The next block then began and was identical to the first block in that a calibration procedure was followed by 24 trials. The calibration results of each block were used for calculating the fixation points of the participants in each individual block. In total, four blocks were run. For each block, the target faces were the same but the foil faces were different and never repeated. Moreover, the blocks in which the foil faces were presented were counter-balanced between subjects such that in each block they were different for different participants. A schematic representation of the experimental design is shown in Figure 1.

Upon completion of the experimental task, participants completed the 13-item revised Cheek and Buss Shyness Scale [22]. Participants were instructed to read each item and decide to what extent it described their behavior. For each item, they selected a number from the following scale: 1 = very uncharacteristic or untrue/strongly disagree, 2 = uncharacteristic, 3 = neutral, 4 = characteristic, 5 = very characteristic or true/strongly agree. The higher the score a participant received, the shyer they were considered.

## Results

### Correlation between Shyness Scores and Accuracy, Correct Response Time, Discriminating Ability d’, and Normalized Criterion c

Prior to running any analyses, d’ and c were calculated by the signal detecting function, choosing target faces as signal and foil faces as noise. Index d’ indicates participants’ ability to distinguish between target and foil faces, diminishing the effect of their tendency to judge the faces as foil or target. Index c indicates the criterion of participants’ judgments: the higher the c is, the stricter participants tend to be in their judgments of the faces as targets.

For all four test blocks collectively examined, the Pearson correlation between participants’ shyness scores and their recognition accuracy, mean response time to the correctly recognized faces (correct response time), discriminating ability d’, and normalized criterion c were computed. All statistical tests were two-tailed unless otherwise specified.

A significant positive correlation was found between participants’ level of shyness and normalized criterion c for Chinese faces (*r = *.51, *p*<.01). Only when recognizing own-race faces (target and foil) did the participants with high shyness levels tend to judge the faces as foils, regardless of whether they were target or foil faces. There were no other significant correlations between participants’ shyness scores and the aforementioned variables, all *p*s >.22.

### Correlation between Shyness Scores and Fixation Proportion on the Eyes, Nose, and Mouth

To examine participants’ fixations on the areas of interest (eyes, nose, mouth), we used a proportion fixation time measure. This measure was obtained by dividing the sum of the fixation time on each of the areas of interest (AOIs) by the total fixation time on the whole face. We first defined a number of AOIs for each face of each race: the whole face (the area within the face contour), the eyes (right and left eyes combined), the nose, and the mouth (See Figure 2). Second, we obtained the total fixation time on each of the AOIs. Third, we computed the proportional fixation time on the AOIs of the eyes, nose, and mouth for each face of each race by dividing the total fixation time on the eyes, nose, or mouth of a particular face by the total fixation time on the whole face. Including both the familiarization block and the following four test blocks, we calculated the mean fixation proportion for each AOI.

Repeated-measures ANOVA analyses were conducted on the participants’ fixation proportion on each AOI for both the learning and reviewing phase and the recognition phase. Shyness level [continuous variable] and face race (own-race, other-race) were chosen as the independent variables. There was a main effect of shyness level for the fixation proportion on the eyes in both the learning and reviewing phase, *F*(1, 27) = 7.48, *p*<.05, *η^2^* = .22, and the recognition phase, *F*(1, 27) = 6.00, *p*<.05, *η^2^* = .18. In all phases, the interaction between face race and shyness level was not significant for participants’ fixation proportion across all AOIs, all *p*s >.05, demonstrating that the relationship between shyness level and scanning patterns did not differ for own- and other-race faces.

**Figure 5:**
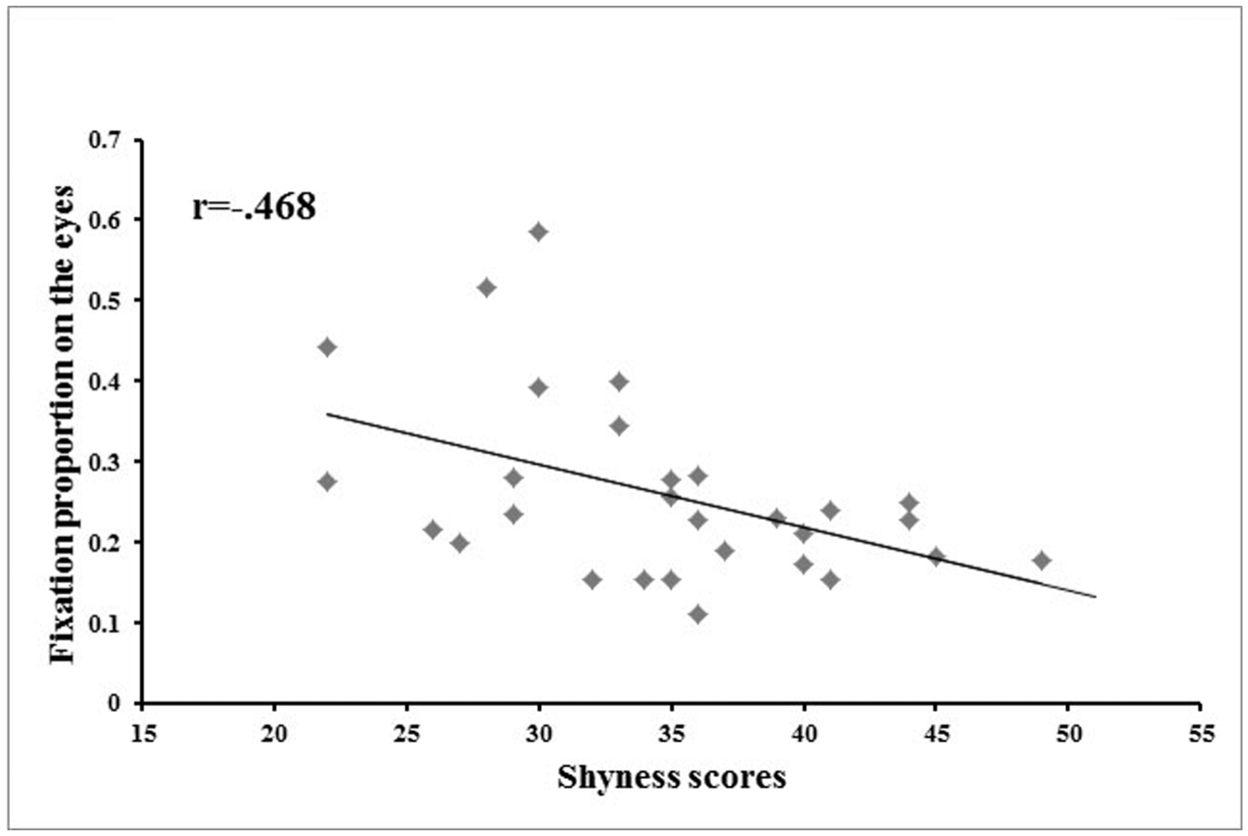
Correlation between shyness and proportion of time spent looking at the eyes during learning **and reviewing.** Scatter plot illustrating the correlation between level of shyness and the proportion of time spent looking at the eyes in the learning and reviewing phase across all three face races.

**Figure 6:**
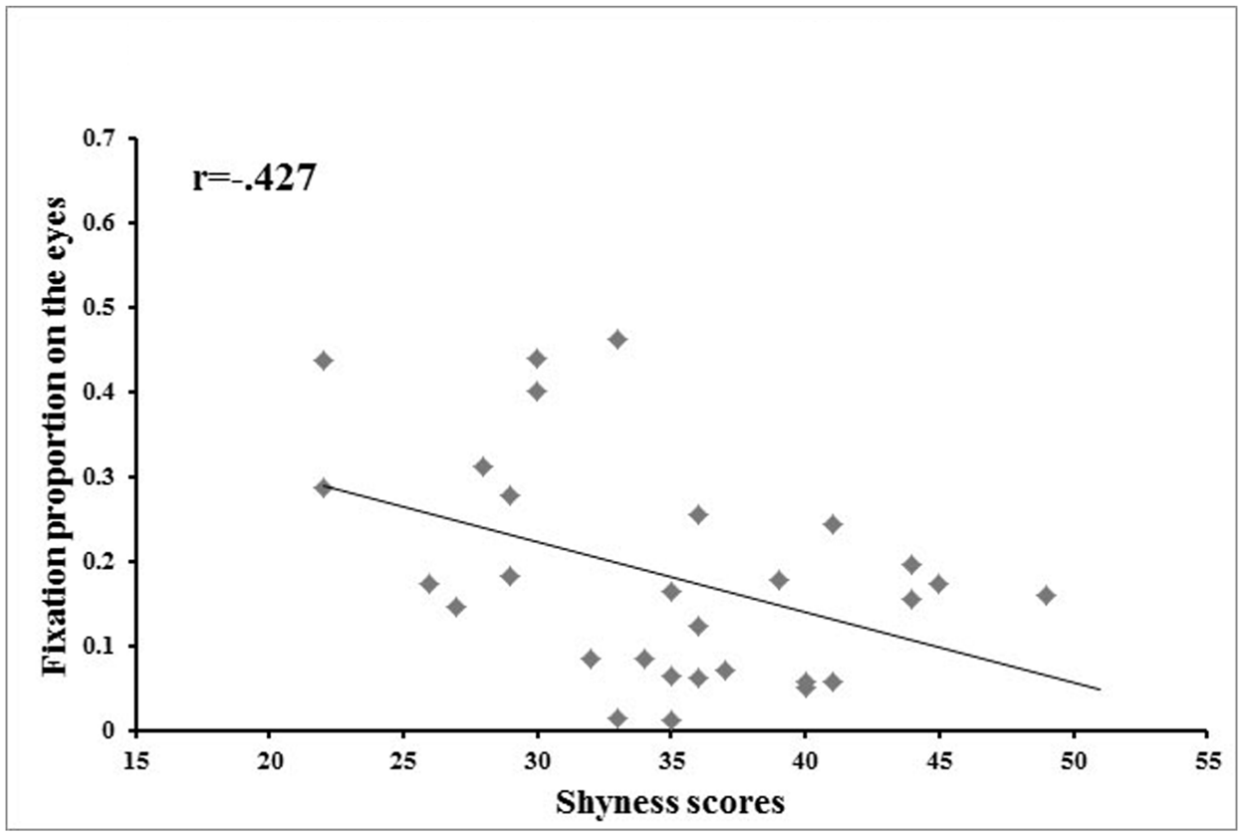
Correlation between shyness and proportion of time spent looking at the eyes during recognition. Scatter plot illustrating the correlation between level of shyness and the proportion of time spent looking at the eyes in the recognition phase across all three face races.

A Pearson correlation analysis revealed that the fixation proportion on the eyes was negatively correlated with participants’ shyness scores when participants were learning, reviewing, or recognizing all three face races. Shyness scores were significantly correlated with fixation proportion on the eyes for Chinese faces in the learning and reviewing phase (*r* = −.40, *p*<.05), Chinese faces in the recognition phase (*r* = −.37, *p*<.05), South Asian faces in the learning and reviewing phase (*r* = −.51, *p*<.01), South Asian faces in the recognition phase (*r* = −.42, *p*<.05), African-American faces in the learning and reviewing phase (*r* = −.44, *p*<.05), and African-American faces in the recognition phase (*r* = −.44, *p*<.05). Overall, the higher a participant’s shyness score, the less he or she looked at the eyes. When all three face races were collectively examined, the correlation between participants’ shyness scores and fixation proportion on the eyes was significant both in the learning and reviewing phase, *r* = −.47, *p*<.05 (Figure 5), and in the recognition phase, *r* = −.43, *p*<.05 (Figure 6).

### Raw Fixation Difference Map between High and Low Shyness Groups

To further explore the fixation data for high versus low shyness participants, we used the iMap Matlab toolbox. We examined the 10 most shy and the 10 least shy participants from our sample based on their overall shyness score. Participants’ fixation data across all face races were analyzed with the iMap method, from which we obtained maps of fixation duration for both the low shyness group and the high shyness group. We also obtained a raw map of the fixation differences between the low shyness and high shyness groups (low shyness - high shyness) in the learning and reviewing phase (Figure 3) and in the recognition phase (Figure 4). All maps are in Z values. Areas showing a significant fixation difference are delimited by white borders (*p*<.05, corrected). In the third column of both Figures 3 and 4, hot colors (i.e., red) denote greater fixations by low shyness participants than high shyness participants and cold colors (i.e., blue) denote greater fixations by high shyness participants than low shyness participants. Values near zero (or white color) indicate similar magnitude in fixation duration between the two categories of participants.

Consistent with the AOI analysis findings, the iMap analysis indicated that participants in the low shyness group fixated more on the eye region than participants in the high shyness group. In particular, low shyness participants appeared to fixate on the pupils of the eyes, whereas high shyness participants appeared to fixate more on the regions just below the eyes.

## Discussion

The results of the present study support our hypothesis that shyness levels influence participants’ scanning patterns in a face recognition task. When given a fixed amount of time to view faces for both familiarization and review, participants’ shyness scores were negatively correlated with the proportion of fixations on the eye region. The shyer the participants were, the less time they spent fixating on the eye region. Even when participants were allowed to terminate their scanning at any point during the recognition phase, the results were similar. This was true not only for viewing own-race faces but also for viewing other-race faces.

The present findings regarding the different scanning patterns between high and low shyness participants cannot be explained by potentially different amounts of time that participants might allocate in recognizing a face or by differences in overall accuracy in face recognition because shyness scores did not correlate with response time and accuracy. Past studies have suggested that the information used to accurately identify faces is largely contained in the eye region [24]. The present findings demonstrate that high shyness participants decreased their fixations on the eye region relative to low shyness participants, which begs the question: Why did the high shyness group not exhibit lower accuracy than the low shyness group in face recognition? As shown in the iMap topographic maps, high shyness participants fixated significantly more than low shyness participants on the regions just below the eyes. Thus, it is possible that high shyness participants processed the information contained in the eyes through their peripheral visual field. Although the high shyness participants’ means of processing information contained in the eyes may have been less efficient than the low shyness group, they were nevertheless capable of meeting the present experimental task’s degree of difficulty.

Our finding that shyness affects participants’ face scanning strategies may be related to the different social intercourse norms between high and low shyness individuals. High shyness or socially anxious individuals often engage in so-called safety behaviors (i.e., avoidance of eye contact) in an attempt to conceal their anxious appearance and avoid negative social outcomes [25]. Thus even when asked to simply view and recognize static facial images, they may continue to apply this strategy because it mimics their behavior in everyday social interactions. These results are consistent with cognitive models of social anxiety [25] and support an avoidance hypothesis to account for high shyness individuals’ tendency to fixate less on the eye region of the face than low shyness individuals. Individuals high in social anxiety tend to focus on the self and avoid potentially threatening information in the environment [26], [27], such as the faces of their peers. Our results are consistent with a number of studies that examine non-clinical social anxiety [13], clinical social anxiety disorders [16], and children with autism [28], [29]. It has been repeatedly shown that individuals with social deficits show decreased looking at the eye region, which may further weaken their social skills because the information used to accurately identify faces is largely contained in this area of the face [24]. Consistent with this idea, shy children’s ability to recognize facial expressions is weaker than non-shy children’s [30], [31], and individuals with avoidant personality disorder are significantly more likely than controls to make errors when classifying fearful expressions [32].

The results of the present study, however, are inconsistent with recent studies that examine social anxiety and face scanning patterns in adults [17] and children [18]. Wieser et al. demonstrated that highly socially anxious women tend to fixate on the eye region of a face longer than borderline and low socially anxious women. It is important to note that Wieser and colleagues used a rectangle to define the AOI in the eye region, which included a larger region around the eyes relative to the AOI defined in our study. Since shy individuals may avoid direct eye contact but still scan the region just below the pupils, we think it is more appropriate to define a smaller AOI in the eye region (just around the eyelids) that includes only the visual information contained in the eyes. This assumption was confirmed by our iMap results in which high shyness participants often fixated just below the lower eyelids, a region that may have been included in Wieser et al.’s specified AOI for the eye region. This difference in the definition of the AOIs may have thus led to the discrepant pattern of results between the two studies.

Brunet et al. [18] found that shyness levels in children were positively correlated with looking time at the eye region. Brunet and colleagues reasoned that shy individuals may be hypersensitive to seeking out evaluative cues that are conveyed by the eyes; thus, such individuals may be more likely to focus on the eye region during face scanning. Our results are counter to this hyper-vigilance hypothesis and instead suggest that shy individuals tend to avoid fixating on the eye region. One reason that may account for the discrepant pattern of results between our study and Brunet et al. is that participant age may influence scanning patterns. Children may find faces with neutral expressions to be especially ambiguous and this ambiguity may lead to heightened vigilance [33]. Thus as shyness levels increase, children may show increased vigilance to the eyes. Because adults have increased experience with neutral faces relative to young children (e.g., individuals often exaggerate their emotional facial expressions when interacting with children), they may be less prone to regard neutral facial expressions as ambiguous and thus neutral expressions may not evoke hypersensitivity in shy adults.

Furthermore, it has been suggested that socially anxious individuals show heightened vigilance to threatening information when they are under a social stressor [27], [34] and that attentional biases are more evident when socially anxious individuals are in a feared social situation [25]. It may be that shy children are more anxious than shy adults when they meet strangers (such as the experimenter), so they experience more stress and anxiety while they are doing experiments relative to adults. Such differences in perceived social pressure and anxiety between children and adults may potentially account for the different pattern of results between our study and Brunet et al.

Our second hypothesis that face race may influence the effect of shyness level on participants’ face scanning patterns was not supported by our results. Regardless of the race of the face stimuli that participants viewed, participants with high shyness levels always fixated less on the eyes than participants with low shyness levels. However, the effect of shyness level on participants’ normalized criterion for judging faces as target or novel was affected by face race. Only when participants were judging own-race faces did their level of shyness significantly correlate with the normalized criterion. That is, only when viewing Chinese faces did the high shyness participants judge the faces as foils more frequently than they judged them as targets. This may be because shy individuals acquire a strict criterion for judging faces as previously seen, so as to not accidentally approach an unfamiliar individual and risk a potentially anxiety-provoking situation. This strategy may be activated automatically only when shy individuals see own-race faces, a category with which they have years of visual experience. Such an interpretation is speculative, however, and future research should examine shy individuals’ criterion for identifying own-race faces in a face recognition paradigm more fully.

The present findings point to several potential avenues for future research. In the present study, we did not submit participants to a social stressor, so it is unclear whether those in the high shyness group might show enhanced vigilance to the eye region when they are given a social stressor. Additionally, the present research is limited in its ecological validity. The faces used in the present study were static, but individuals always scan faces in communicative dynamic contexts. While it is difficult to study such aspects in the laboratory setting, future studies should attempt to incorporate some aspects of dynamic communication, such as the effect of positive and negative expressions, on face scanning. Since some previous studies have shown that socially anxious individuals tend to show increased vigilance to emotional faces [27], [35], it is meaningful to explore the influence of shyness on participants’ scanning of emotionally expressive faces. Lastly, it is possible that the different scanning patterns observed between shyness groups may reflect the different social intercourse norms between high and low shyness individuals in everyday life; however, there may be other important differences between these two groups when they are scanning faces. One possibility is that the two groups might experience different emotional states when they participate in psychological experiments. Additional studies with the same design as ours but involving an examination of participants’ emotional states would address this issue, which in turn would further elucidate the nature of the differences between high and low shyness groups in their scanning of faces.

In summary, our results support an avoidance hypothesis in which shy individuals tend to fixate less on the eye region of human faces relative to non-shy individuals. The correlation between shyness level and scanning patterns does not differ for own- and other-race faces, suggesting that prolonged visual experience with a category of faces does not moderate the effect. Identifying differences in scanning patterns between high shyness and low shyness groups is crucial if we are to understand the attentional mechanisms that underlie the various processing strategies employed by socially anxious individuals. Such research can ultimately aid in the development of more efficient cognitive treatments for those with social anxiety and motivate future studies to examine the way in which individual differences influence facial scanning patterns.
